# Individualized dosing of oral propranolol for treatment of infantile hemangioma: a prospective study

**DOI:** 10.11604/pamj.2019.32.155.16760

**Published:** 2019-04-08

**Authors:** Arun Prasad, Amit Kumar Sinha, Bindey Kumar, Abhiranjan Prasad, Manju Kumari

**Affiliations:** 1 Department of Pediatrics, All India Institute of Medical Sciences, Patna, India; 2 Department of Pediatric Surgery, All India Institute of Medical Sciences, Patna, India; 3 Department of General Surgery, Anugrah Narayan Magadh Medical College, Gaya, India; 4 Department of Pharmacology, Jawahar Lal Nehru Medical College, Bhagalpur, India

**Keywords:** Infantile hemangioma, propranolol, dose, side effects

## Abstract

**Introduction:**

infantile hemangioma is the most common benign tumor in infancy. Currently, oral propranolol is the treatment of choice for infantile hemangioma, but there is no consensus when it comes to its recommended dosage for this condition. Hence this study was conducted to find out the appropriate dosage of oral propranolol for treatment of infantile hemangioma.

**Methods:**

A prospective study was conducted on 25 patients with infantile hemangioma, who were treated with gradually increasing dose of propranolol starting from a lower dose of 1mg/kg/day.

**Results:**

17/22(76%) patients showed regression of the tumor at the dose of 1- 1.5 mg/kg/d. 5/22(24%) patients were unresponsive to the treatment with the lower dose and they did not respond even with the gradually escalated dose of 3-4 mg/kg/day.

**Conclusion:**

Propranolol in a lower dose of 1-1.5 mg/kg/day is safe and efficacious in the treatment of infantile hemangioma and the lesions which do not show initial response to the lower dose are unlikely to respond to the higher dose of 3-4 mg/kg/day.

## Introduction

Infantile hemangiomas are the most common infantile tumor [1, 2]. The prevalence of Infantile hemangioma in mature neonates is around 4.5% with a female (2.3-2.9 times higher) and white predominance [3]. The predominant locations are head and neck [4]. They arise in initial few weeks of life and then display a period of active growth followed by spontaneous involution. The proliferative phase spreads over three to six months. Most Infantile hemangiomas do not require therapy and regress spontaneously over months to years. However, about 10-15% of the cases result in complications such as obstruction, ulceration and disfigurement. It may also carry risk of bleeding. Treatment is required in such conditions [1]. Therapeutic effect of Propranolol over Infantile hemangioma was detected incidentally in the year 2008, when regression of facial hemangioma was noted in a child while being treated for hypertrophic cardiomyopathy by this molecule [5]. Since then, it is being used for infantile hemangioma and currently oral Propranolol is the treatment of choice for this condition [6]. However, there is lack of consensus over its dosage as cited in literature of different studies [7-9].

Propranolol has been used for cardiovascular indications in the dosage up to 4mg/kg/d, but for treatment of infantile hemangioma, a lower dose is required. For treatment of Infantile hemangioma, some studies favor a lower dose (1-1.5 mg/kg/dose) [7, 8], while others favor a higher dose (3mg/kg/day) [9]. The precise mechanism of action of Propranolol in the treatment of infantile hemangioma is unclear. The possible mechanisms include vasoconstriction, inhibition of angiogenesis and induction of apoptosis [6]. Although Propranolol has been widely used for this indication, other Beta blockers like Nadolol [10-13] and Acebutolol [14] have also been shown to be effective for Infantile hemangioma in small non-controlled studies. Other agents with reported activity in treating Infantile hemangioma include corticosteroids, Interferon alpha and Vinca alkaloids [15]. Besides pharmacotherapy, other treatment modalities include Laser therapy and surgical resection. Sometimes a combination of these modalities is required [16]. We conducted a prospective cross sectional study in 25 cases of infantile hemangioma to find out appropriate dosage of oral Propranolol for treatment of this condition.

## Methods

Twenty seven patients of age 2 months and older, with vascular lesions suggestive of infantile hemangioma on clinical grounds, were selected. Approval from ethics committee of the institute was taken. History of any hypoglycemic event with the child was enquired. History of any heart block in the mother was also enquired in view of its potential association with complete heart block in the child. Baseline heart rate & blood pressure were noted. ECG and echocardiography were done to rule out any conduction or structural abnormality. Ultrasonography of the vascular lesions was done to confirm the diagnosis of infantile hemangioma. Abdominal ultrasound was also done to look for any hemangioma inside the abdomen. One patient was lost to follow up during initial investigations. Lesion of another patient came out to be venous malformation on ultrasonography, hence excluded. Amongst the remaining 25 patients, there were 18 males and 7 females. It was planned to start oral propranolol in patients with treatment indications, at the dose of 1 mg/kg/day with gradual increment by 0.5 mg/kg/d in subsequent visits. Daily dose of this medicine was given in two divided doses. First follow up was planned after 1 week, thereafter every 2 week till any response was observed or full dose of 3-4 mg/kg/d was reached. Indications for starting propranolol therapy were functional impairment, risk of bleeding and aesthetic concerns. Lower respiratory tract infection related bronchospasm was noted in two infants and they were treated before starting propranolol. Exclusion criteria were previous treatment with propranolol, rhythm abnormality like complete heart block, ventricular systolic dysfunction and any structural cardiac abnormality like ASD/VSD or PDA. 25 patients were selected for starting oral propranolol therapy, based on the above indications. 24 patients were given oral propranolol with starting dose of 1 mg/kg/d, as OPD treatment.

One patient aged 2 months was admitted for 1 day for beginning oral propranolol and observation of any side effects. Caregivers of the child were advised to ensure regular feeding of the child to avoid hypoglycemia and to report back anytime in case of any adverse event, possibilities of which were explained to them. On follow up visit, response to the treatment was evaluated. Any fading of color of the tumor based on comparison with the previous photograph (in case of superficial hemangioma) or any decrease in size of the lesion were assessed. Blood pressure and heart rate were checked to look for any side effects like hypotension and bradycardia. Patients who tolerated the propranolol well, were given increased dose, 1.5 mg/kg/day after one week. Two patients with superficial hemangioma were noted to have marked response on follow up after 1 week in the form of marked fading of the color of hemangioma and reduction in its size and it was decided to continue with the same dose of 1 mg/kg/day in them for further duration of therapy. 2 infants developed irritability within 1 week of starting therapy and propranolol was discontinued in them. Others were treated as per plan. Those who responded to therapy with fading of color of tumor as compared to previous photograph, softening in consistency of the tumor or reduction in its size on physical measurement, were continued on same dose and a monthly follow up was done subsequently. Response for the deep (subcutaneous) hemangioma was assessed by physical measurement of the tumor. For this study, response was defined as 'subsided' when there was ≥ 90% reduction in size of tumor and 'partial' when reduction in tumor size was < 90%. Lei chang *et al.* used the similar parameter of measurement for labeling hemangioma as 'regression' and 'partial regression' after propranolol therapy [17]. In non-responders, the dose of propranolol was gradually increased at the rate of 0.5mg/kg/d every month till any response was observed or the maximum dose of 3-4mg/kg/dose was achieved. If no response was noted, one month after the maximum dose, propranolol was discontinued in them. In responder group, the criteria for tapering off propranolol was decided on the basis of subsidence of the tumor, decrease in tumor size and keeping the same size for 3 consecutive months and the child has received a minimum of 6 months of therapy. Follow up was done for 6 months after completion of therapy.

## Results

**Figure 1:**
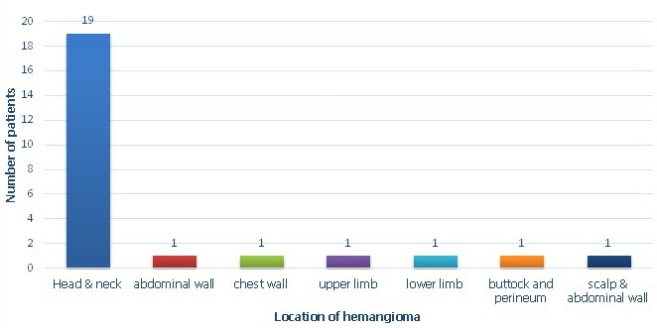
Site of infantile hemangioma in 25 patients

**Figure 2:**
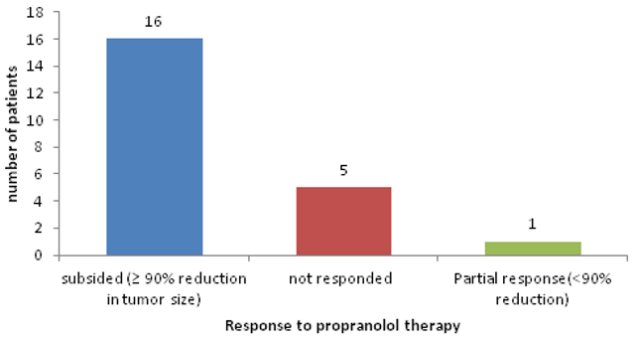
Effect of propranolol therapy on infantile hemangioma

**Figure 3:**
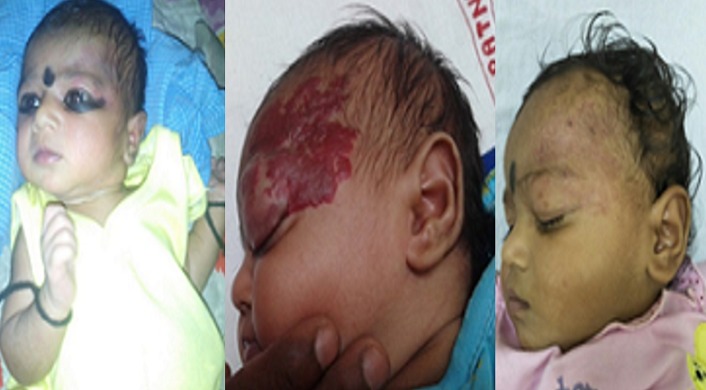
A child at various stages (before and after hemangioma) -10 days of life, 2 months of life and 9 months after propranolol

**Figure 4:**
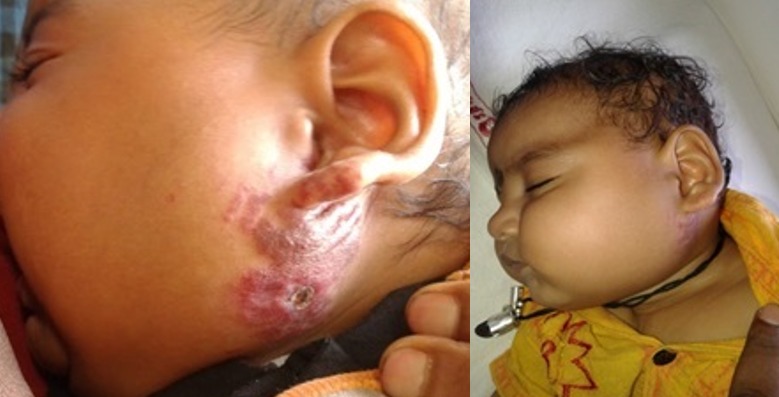
Superficial hemangioma before and after treatment

**Figure 5:**
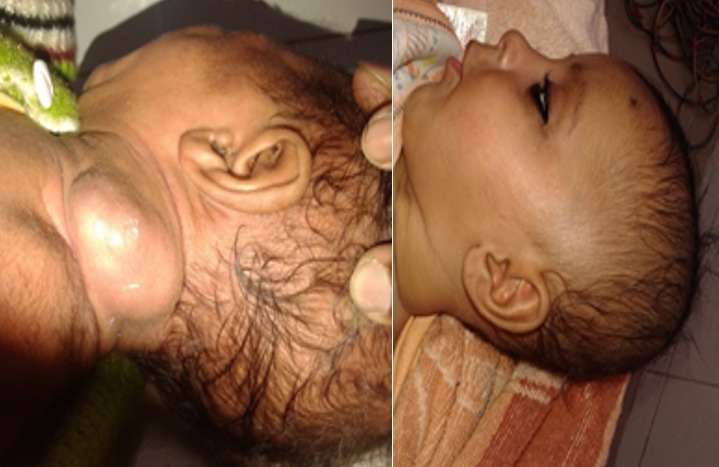
Deep hemangioma before and after treatment

**Figure 6:**
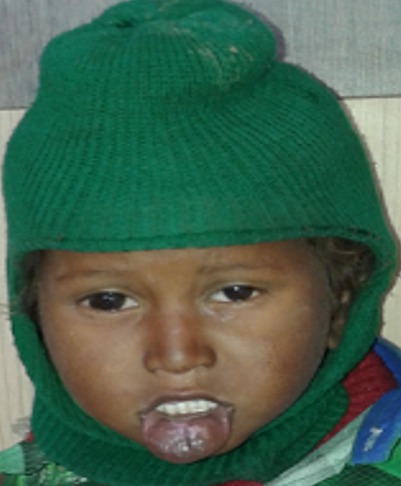
Hemangioma of lip unresponsive to propranolol therapy

25 patients were started on oral Propranolol for treatment of their Infantile hemangioma. There were 18 males and 7 females (Table 1). Head and neck region was predominantly involved (76%) with infantile hemangioma (Figure 1). Two infants developed irritability during first week of therapy and lost to follow up. 1 patient lost to follow up after 1 month of treatment. 22 patients were further continued with the treatment. 17/22 (77%) patients responded to the treatment with regression of the tumor size (Figure 2, Figure 3, Figure 4, Figure 5, Figure 6). Median dose of Propranolol in the responder group was 1.5 mg/kg/day. 1/22(5%) patient showed partial response. Response rate was maximum 93% (15/16) in children of ≤ 1 year age group (Table 2). 5/22(24%) patients did not respond to the treatment (Figure 2). Response to treatment in superficial hemangioma in the form of color change was noted within 1 week of starting therapy. Average duration of therapy was 10 months. 5 patients, who did not respond to the lower dose, were given gradually escalated dose of 3-4 mg/kg/day (4 patients received 3mg/kg/d & 1 Patient received 4 mg/kg/day). They were evaluated after 1 month after starting this maximum dose and when no response was observed in them, they were discontinued Propranolol therapy. None of the 22 patients (even one who was given 4mg/kg/d of Propranolol), were found to have bradycardia. 5/13 cases of superficial hemangioma developed ulceration during therapy and they were managed with topical Mupirocin. Two patients had an unusual side effect of itching over lesion after beginning of oral Propranolol. They were managed with two weeks of oral Hydroxyzine. Besides irritability in two infants and local ulceration over the lesion in 4 children, no significant side effects were noticed (Table 3). Follow up was done for 6 months after completion of therapy. 2/21 (10%) patients showed recurrence of lesion after completion of therapy. They were 10 months and 11 months old after completion of 6 months and 9 months of therapy respectively. They responded with further extending the duration of therapy for 6 months after restarting with the previous dose of 1.5 mg/kg/day.

**Table 1:** Age and sex distribution of infantile hemangioma (n=25)

Age group	Male	Female	Total (%)
≤1 year	13	5	18(71%)
>1 year- 5 Year	4	1	5 (19%)
>5 years	1	1	2 (9%)
Total	18	7	25

**Table 2:** Response to propranolol therapy in different age group (n=22)

Age Group	Total No.	Response	No Response
≤1year	16	15	1
>1Year -5 Year	4	2	2
>5year	2	-	2

**Table 3:** Side effects of propranolol therapy (n=25)

Side effects	Number (%)
None	17 (68)
Ulceration over lesion	05 (20)
Irritability	02 (08)
Itching over lesion	02 (08)
Hypotension	00
Bradycardia	00

## Discussion

After the incidental discovery of therapeutic effect of oral propranolol in treatment of infantile hemangioma, it has become the treatment of choice [18]. Most of the superficial hemangioma responded to the lower dose of propranolol (1.5mg/kg/dose) and those who responded, showed response within one week of starting treatment. Maximum response was noted in infants (< 1 year age group). Ren W *et al* (2017) also described effectiveness of low dose propranolol (1-1.5mg/kg/day) with change in color and growth of the tumor within one week [7]. Gabor Katona *et al* (2012) described resembling findings of the effect of propranolol used in the dosage of 2mg/kg/day, as regression in tumor size in first week [19]. After initial regression, the later improvement is much slower, sometimes with periods of stagnation. The treatment should be continued for at least 6 months because early cessation can cause a relapse [19]. Cessation of therapy before 1 year of age also may be associated with a relapse [20, 21].

Ultrasonography is a good tool for objective assessment of the change in tumor size [22]. Leaute-Labreze *et al.*conducted a controlled trial with 1mg/kg/d and 3 mg/kg/d of oral propranolol with approximately 100 patient in each group. They found complete or near complete resolution of the hemangioma after six months of oral propranolol in 50% of the patients in 1 mg/kg/d group as compared to 60% in 3 mg/kg/day group, but the side effects like hypotension was more common in 3mg/kg group (3% vs 1%). Similarly Bronchospasm was more common in 3 mg/kg group compared to 1 mg/kg group (1% vs 0%) [23]. They observed that agitation as a side effect is more common in 1 mg/kg group (18%) as compared to 3 mg/kg(8%) group. This means that this side effect of propranolol is dose independent. We also observed irritability in 8% of children after starting propranolol therapy. This is important to explain to the caregiver of the child regarding possible side effects of propranolol before starting treatment. Lei Chang *et al.* described that there is individual variation of response after administration of propranolol. Chinese subjects have at least two fold greater sensitivity to propranolol [17]. We also suggest that the dose of propranolol should be individualized. A lower dose should be considered for therapy, if the response is observed with the lower dose. The non-responders who were labeled to have hemangioma on the basis clinical grounds and ultrasonography, might have had anatomically or histologically different lesion. We have found recurrence in 2 infants when the propranolol therapy was tapered off before 1 year of age. Thus the possibility of recurrence should be a point of consideration while tapering off propranolol therapy.

Recommendations: for the treatment of infantile hemangioma, dose of propranolol should be individualized and started with a lower dose with gradual escalation. If response is achieved with the lower dose (1-1.5 mg/kg/dose), further treatment should be continued with the lower dose. Those who show a marked response after initiation of therapy at the dose of 1 mg/kg/day, should be continued with the same dose till regression of the tumor size. If response is not achieved 1 month after the maximum dose (3-4mg/kg/day), it is unlikely that infantile hemangioma will respond to this pharmacotherapy and it should be discontinued to consider other modalities of treatment. Most of the patients can be managed as outpatient. Parents should be explained regarding possible side effects of propranolol including hypoglycemia and should be advised that the child needs to be fed regularly. They should also be explained that in case of any adverse event, they should discontinue propranolol and take medical consultation in nearby health facility. Inpatient management has been advocated for infants and children with - corrected gestational age of ≤ 8weeks, inadequate social support, comorbid conditions affecting the cardiovascular system, symptomatic airway hemangioma and history of hypoglycemia. As recurrence is more common if propranolol is stopped before 6 months of treatment or before 1 year of age of the child, it is logical to use lower dose for a longer period rather than higher dose for a shorter period.

## Conclusion

Oral propranolol in the lower dose of 1-1.5 mg/kg/day is safe and efficacious in treatment of infantile hemangioma. Those who do not show initial response to the lower dose, remain unresponsive even at higher dose of 3-4 mg/kg/day. Most of the children can be treated as outpatient after proper counselling regarding possible side effects of propranolol.

**Limitations of the study:** Although we suggest individualized and lower dose of propranolol therapy as per findings of this study, the limitations of this study are that this is an open labeled study and sample size is small.

### What is known about this topic

- Oral propranolol is efficacious in treatment of infantile hemangioma;
- Low dose propranolol (1-2 mg/kg/day) as well as higher dose propranolol (3mg/kg/d) has been used to treat this condition;
- Recurrence of hemangioma after the treatment is a known complication if propranolol is stopped before 1 year of age.

### What this study adds

- Dose of oral propranolol in treatment of infantile hemangioma should be individualized. Treatment should be started with the low dose (1-1.5 mg/kg/d) and if the response is observed on a low dose, the same may be continued for further course of therapy. It is advisable to continue this pharmacotherapy beyond 1 year of the age to avoid recurrence.
- Infantile hemangioma which does not show initial response to the lower dose (1-1.5mg/kg/day) of propranolol is unlikely to respond to its higher dose (3-4 mg/kg/d). Older children are less likely to respond with oral propranolol therapy for their infantile hemangioma. Other modalities of therapy should be considered in them.

## Competing interests

The authors declare no competing interests.
